# Green- and Blue-Emitting Tb^3+^-Activated Linde Type A Zeolite-Derived Boro-Aluminosilicate Glass for Deep UV Detection/Imaging

**DOI:** 10.3390/ma17030671

**Published:** 2024-01-30

**Authors:** Yongneng Xiao, Shaoyi Hou, Zhenhuai Yang, Xingxing Huang, Yuanjun Guo, Siyu Ji, Xiaochan Huang, Fengshuang Wang, Qiang Hu, Xiaodong Guo

**Affiliations:** Jihua Laboratory, Foshan 528251, China; xiaoyn@jihualab.com (Y.X.); housy@jihualab.com (S.H.); huangxx@jihualab.com (X.H.); guoyj@jihualab.com (Y.G.); jisy@jihualab.com (S.J.); huangxc@jihualab.com (X.H.); wangfs@jihualab.com (F.W.); huqiang@jihualab.com (Q.H.); guoxd@jihualab.com (X.G.)

**Keywords:** boro-aluminosilicate glasses, luminescence, Tb^3+^, phosphor, UV detection and imaging

## Abstract

Tb^3+^-activated LTA zeolite-derived boro-aluminosilicate glass samples with a composition of xTb_2_O_3_-68(Na_2_O-Al_2_O_3_-SiO_2_)–32B_2_O_3_ (x = 0.2, 1.0 and 10 extra wt%) were prepared using the melt-quenching method. The emission spectra recorded upon ultraviolet (UV) excitation with two different wavelengths of 193 and 378 nm showed blue light (^5^D_3_ to ^7^F_J=6,5,4_ and ^5^D_4_ to ^7^F_6_ transitions of Tb^3+^) and green light (^5^D_4_ to ^7^F_5_ transition of Tb^3+^) emissions with comparable intensities up to a Tb^3+^ concentration of 10 extra wt%. Of note, the mean decay times of the green luminescence of the glass samples were relatively fast (<20 μs). The synthesized glass has potential in applications concerning UV imaging, UV detection, and plasma display panels.

## 1. Introduction

There is ongoing research on the luminescence properties of rare earth-doped glasses and ceramics [1,2] potentially suitable for applications concerning lighting [3], displays [4,5], X-ray scintillation [6], increasing the efficiency of solar cells [7,8], and UV detection and imaging [9]. Emission in such materials is the result of optical transitions attributed to energy levels of 4f-electrons of rare earth ions (e.g., 4f^8^ (for Tb^3+^), 4f^7^ (for Gd^3+^), 4f^6^ (for Eu^3+^), and 4f^12^ (for Tm^3+^)) [10]. Thus, for the case of Tb^3+^-doped materials, the emission is due to transitions from ^5^D_3_ (violet-blue light) and ^5^D_4_ (green-orange light) levels to integer multiplets of ^7^F_J_ of Tb^3+^ ions [11]. Additionally, Tb^3+^ is an important dopant in commercial phosphor LaPO_4_:Ce^3+^, Tb^3+^ [12,13]. Nevertheless, in practice, the emission spectra of Tb^3+^-doped materials are often dominated by a characteristic green light (543 nm; ^5^D_4_ to ^7^F_5_ transition) due to a non-radiative relaxation from the ^5^D_3_ level to the ^5^D_4_ level via cross-relaxation to a neighbor Tb^3+^ [11,14,15,16]. In fact, examples of Tb^3+^-doped glasses showing ^5^D_3_-originated blue emission with intensities comparable to those of the green emission are limited to glasses with low contents of Tb^3+^ (typically no more than 1 mol%) [14,15,17,18,19]. The green luminescence decay time of Tb^3+^-doped glasses is typically on the order of ms [16,20,21,22,23,24,25,26], which normally does not satisfy the fast response time required for some applications, e.g., scintillation detectors [27] and plasma display panels [28,29]. In fact, examples of Tb^3+^-doped glasses with decay times on the order of μs are rare [29].

Different host lattices have been used for Tb^3+^-activated glasses, e.g., aluminates [14,19,21,26,30], phosphates [16,18,22,23], and germanates [24,25,31]. Linde Type A (LTA) zeolites are synthetic aluminosilicates with a porous and periodic structure with the composition of Na_12_(AlSi)_12_O_48_⋅27H_2_O, which can adopt an amorphous structure via thermal treatment at high temperatures [32]. To our knowledge, only a few studies have examined the luminescence properties of rare earth-activated LTA zeolite-derived ceramics/glasses, which have included ions of Tb^3+^ [20], Dy^3+^ (with or without Ag^+^) [33], and Eu^2+^ [20,34]. In the study that used Tb^3+^ as the dopant [20], the sample was prepared by ion exchanging Tb^3+^ for Na^+^ in the LTA zeolite in deionized water, followed by applying the melt quenching method. The sample was green-emitting and showed a green luminescence decay time of 2.398 ms. Thus, in the present paper, we report on the preparation, physical characterization, and luminescence study of green- and blue-emitting Tb^3+^-doped LTA zeolite-derived boro-aluminosilicate glasses that show fast green luminescence decay times on the order of μs.

## 2. Experimental Processes

### 2.1. Sample Preparation

**Figure 1:**
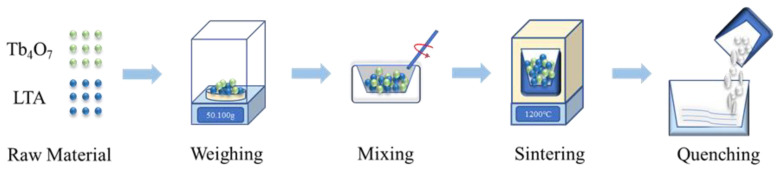
Schematic diagram of the preparation process of LTA:Tb^3+^ glass samples.

The Tb^3+^-doped glass samples were prepared via the melt quenching method using LTA zeolites (4A, A. R, Tosoh, Tokyo, Japan; Na_12_(AlSi)_12_O_48_⋅27H_2_O), boron oxide (B_2_O_3_, 98%, Shanghai Aladdin Biochemical Technology Co. Ltd., Shanghai, China), and Tb_4_O_7_ (Shanghai Diyang Chemicals Co., Ltd., Shanghai, China). The amounts of LTA zeolite and boron oxide were fixed at 34 g (68 wt%) and 16 g (32 wt%), respectively. Thus, to 50 g mixtures of LTA zeolite and boron oxide, Tb_4_O_7_ was added in amounts of 0.1 g (extra 0.2 wt%; sample #1), 0.5 g (extra 1.0 wt%; sample #2), and 5 g (extra 10 wt%; sample #3). The powder mixtures were placed in an agate mortar and mixed homogeneously. Heating the mixture to 1200 °C using a high-temperature muffle furnace [35], the Tb^3+^ ions diffuse and evenly distribute within the LTA molecular sieve. Subsequently, the thoroughly melted liquid is quenched by pouring it into water, thus obtaining colorless transparent glass with the nominal composition xTb_2_O_3_-68(Na_2_O-Al_2_O_3_-SiO_2_)-32B_2_O_3_ (x = 0.2, 1.0 and 10 extra wt%). We have also illustrated a brief description of the preparation process [36,37,38,39] of LTA:Tb^3+^, as shown in Figure 1.

### 2.2. Characterization

The structures of all of the obtained samples were analyzed with X-ray diffraction (XRD) (Rigaku, Model Mini Flex 600, Tokyo, Japan), using Cu K_α_ irradiation (λ = 1.5418 Å) operated at 40 kV, 15 mA. The morphology of the fracture surfaces of the samples was observed using a scanning electron microscope (SEM), while energy dispersive X-ray spectroscopy (EDX) was performed in the SEM (Verios 5 UC, Eindhoven, The Netherlands). The optical transmission spectra were recorded with an ultraviolet–visible–NIR spectrophotometer (PerkinElmer, LAMBDA 1050, Waltham, MA, USA). The steady photoluminescence (PL) and photoluminescence excitation (PLE) spectra and the dynamic emission decay curves were recorded using a fluorescence spectrophotometer (Edinburgh Instruments, FLS-1000, Livingston, UK).

## 3. Results and Discussion

The SEM images did not show pores in the samples. The results of the experimental determination of the wt% of the elements in the samples using EDX are presented in Table 1. The values in Table 1 are the average values of two measurements. The inconsistencies between the nominal and experimental compositions can be explained by (i) the uncertainty in the determination of wt% of the elements (18–25%) dictated by the instrumentation used, and (ii) the fact that the glass matrix consisted of light elements such as B, Al, Si, Na, and O, which could be evaporated from the surface, e.g., B could have been undetected due to being very light.

**Figure 2:**
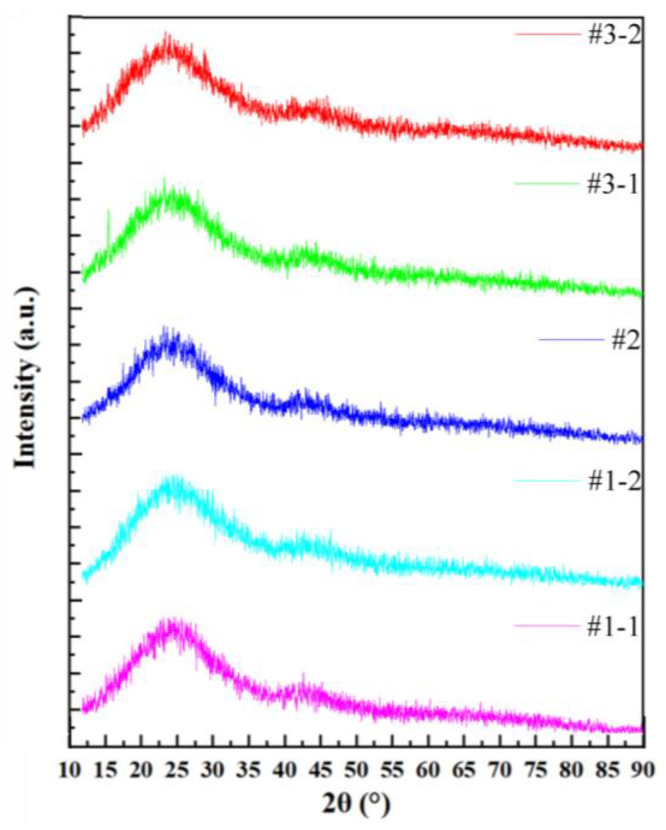
XRD analysis of the Tb^3+^-activated LTA zeolite-derived boro-aluminosilicate glass samples.

**Figure 3:**
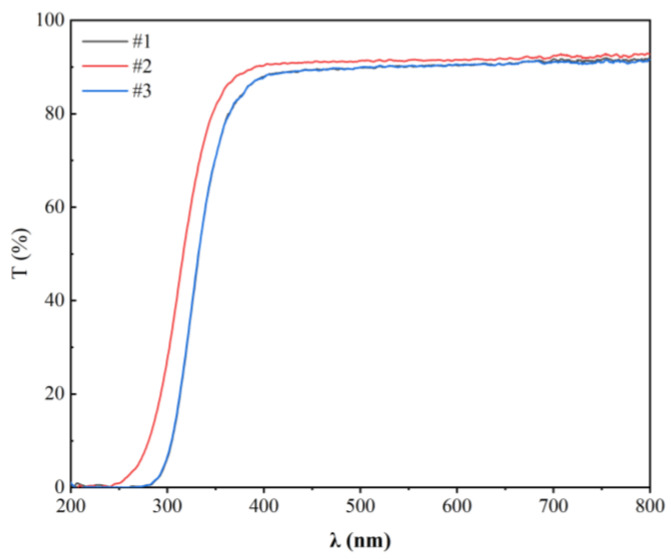
Transmission spectra of the Tb^3+^-activated LTA zeolite-derived boro-aluminosilicate glass samples.

**Figure 4:**
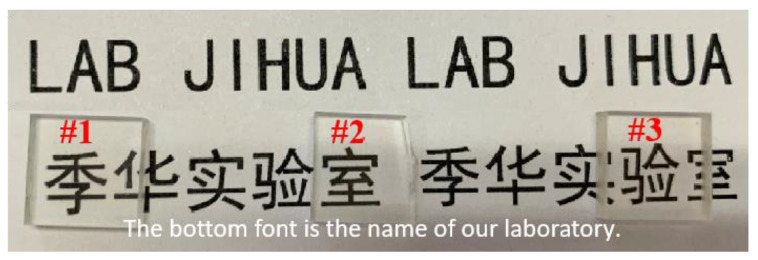
Photos of the Tb^3+^-activated LTA zeolite-derived boro-aluminosilicate glass samples (The bottom font is the name of our laboratory).

**Figure 5:**
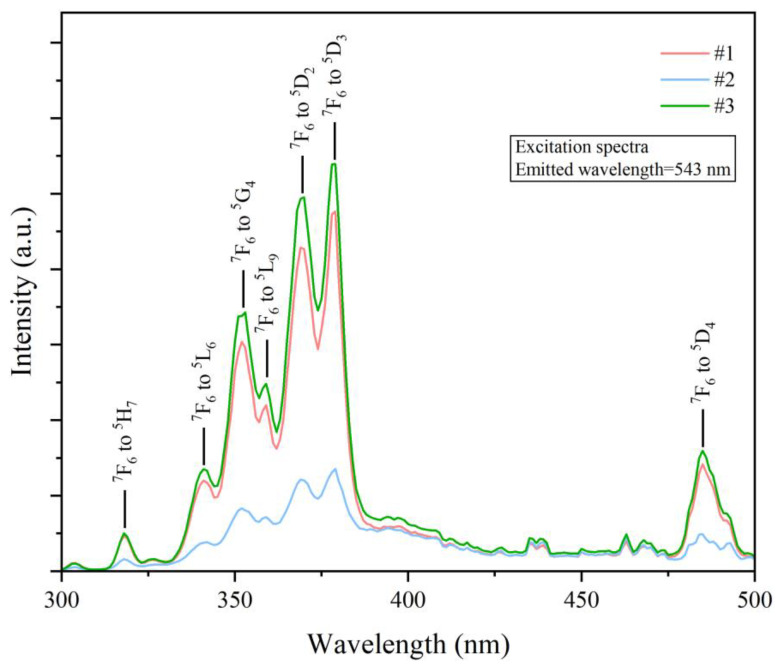
PLE spectra of the Tb^3+^-activated LTA zeolite-derived boro-aluminosilicate glass samples (λ_em_ = 543 nm).

The XRD analysis of samples #1, #2, and #3 did not show crystalline peaks, thus confirming the glass nature of the samples (Figure 2). The transmission spectra of samples #1, #2, and #3, presented in Figure 3, showed that the visible light transmittance was beyond 85%. Also, photos of these samples were colorless and transparent (Figure 4). The transmittance of a standard sample showed absorption peaks at 378 nm and 485 nm, which were attributed to ^7^F_6_ to ^5^D_3_ and ^7^F_6_ to ^5^D_4_ transitions of Tb^3+^, respectively [30,31]. The high transparency of the fabricated LTA zeolite glass samples in the visible light range and the low doping concentration of Tb^3+^ could have resulted in overshadowing or overlapping of the absorption peak of Tb^3+^ by those of the LTA base material in the absorption spectra. Therefore, the transmittance spectra of the Tb^3+^:LTA material did not show a distinct absorption peak for Tb^3+^, and there was no significant correlation with the doping amount of Tb^3+^. The peaks observed and the corresponding transitions in the PLE spectra (λ_em_ = 543 nm) were 304 nm (^7^F_6_ to ^5^H_6_), 318 nm (^7^F_6_ to ^5^H_7_), 341 nm (^7^F_6_ to ^5^L_7_), 354 nm (^7^F_6_ to ^5^L_9_), 369 nm (^7^F_6_ to ^5^D_2_), 378 nm (^7^F_6_ to ^5^D_3_), and 485 nm (^7^F_6_ to ^5^D_4_) (Figure 5) [30,31].

**Figure 6:**
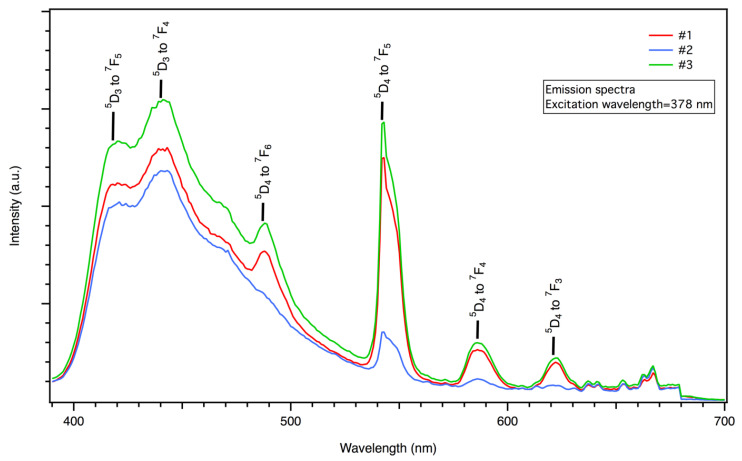
PL spectra of the Tb^3+^-activated LTA zeolite-derived boro-aluminosilicate glass samples (λ_exc_ = 378 nm).

**Figure 7:**
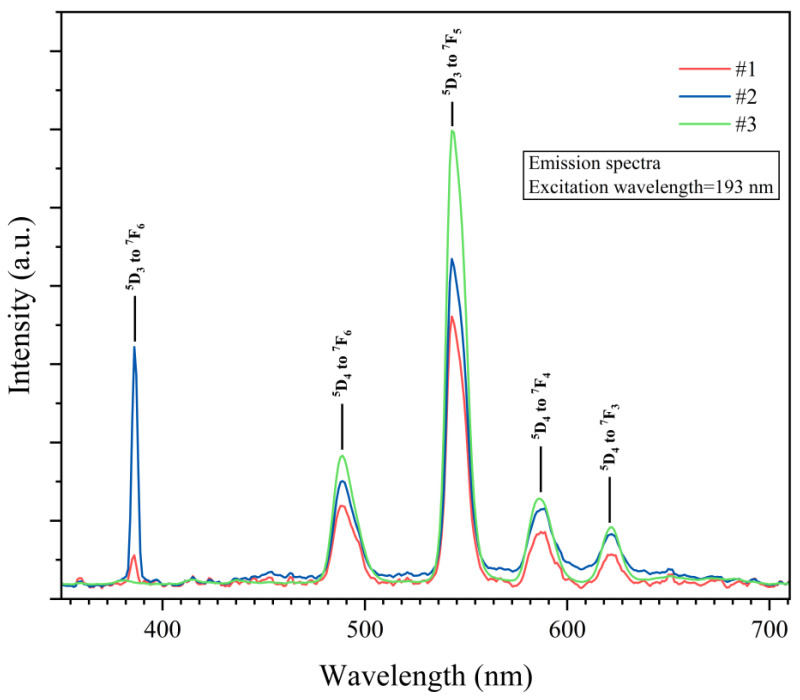
PL spectra of the Tb^3+^-activated LTA zeolite-derived boro-aluminosilicate glass samples (λ_exc_ = 193 nm).

**Figure 8:**
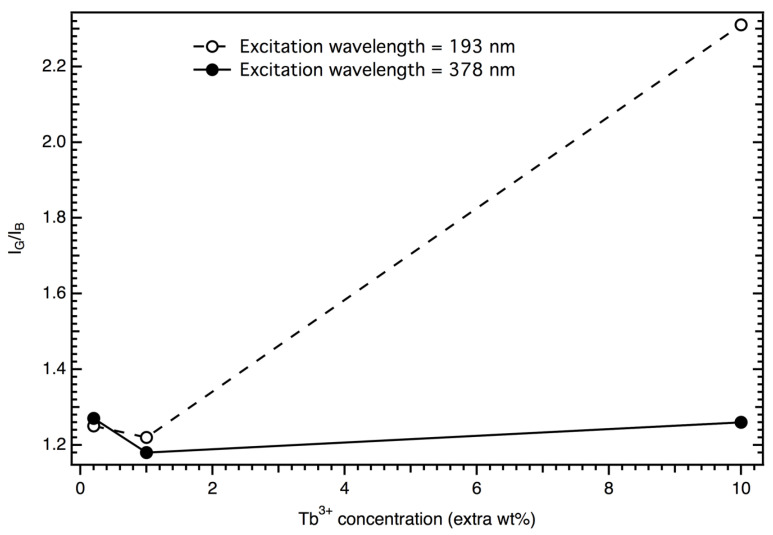
Variation in I_G_/I_B_ with varying concentrations of Tb^3+^ (I_G_ and I_B_ were determined by integrating the intensities of the green and blue peaks in the emission spectra, respectively).

The PL of the samples was examined by measuring their emission spectra upon excitation with two ultraviolet (UV) light excitations, one with a wavelength of λ_exc_ = 378 nm, which lay in the ultraviolet A (UV-A)/near-ultraviolet (N-UV) regions, and another with a wavelength of λ_exc_ = 193 nm (a laser light), which lay in the ultraviolet C (UV-C)/far-ultraviolet (F-UV) regions. Peaks of both ^5^D_3_ and ^5^D_4_ transitions were present in the emission spectra upon excitation with UV-A/N-UV light, which were located at 418 nm (^5^D_3_ to ^7^F_5_), 440 nm (^5^D_3_ to ^7^F_4_), 487 nm (^5^D_4_ to ^7^F_6_), 543 nm (^5^D_4_ to ^7^F_5_), 586 nm (^5^D_4_ to ^7^F_4_), and 621 nm (^5^D_4_ to ^7^F_3_) [16,30,36] (Figure 6). Upon excitation with UV-C/F-UV light, the same peaks from the ^5^D_4_ to ^7^F_J_ transitions were present, but emission from ^5^D_4_ to ^7^F_J_ was limited to one peak at 386 nm (^5^D_4_ to ^7^F_6_) [14] (Figure 7). In all of these transitions, ^5^D_4_–^7^F_6_ (487 nm) and ^5^D_4_–^7^F_5_ (543 nm) are magnetically dipole and parity-forbidden transitions, respectively [20]. Therefore, LTA:Tb^3+^ exhibits a strong emission intensity at 543 nm. In this context, we should point out that a non-radiative relaxation from the ^5^D_3_ to the ^5^D_4_ levels via cross-relaxation to a neighbor Tb^3+^ is a well-known phenomenon in Tb^3+^ systems [11,14,15,16]. This process occurs because due to the closely matched energy difference between the ^5^D_4_ and ^5^D_3_ levels (5800 cm^−1^) and the ^7^F_6_ and ^7^F_0_ levels (5700 cm^−1^), excitation from ^7^F_6_ to ^7^F_0_ promotes the non-radiative drain from ^5^D_3_ to ^5^D_4_ of a nearby ion ((^5^D_3_, ^7^F_6_) → (^5^D_4_, ^7^F_0_)) [11,14,15]. Thus, if the dispersion of Tb^3+^ in the matrix is ideal, the ratio of the intensity of the green light to the intensity of the blue light (I_G_/I_B_) is expected to increase when Tb^3+^ concentration is increased [15]. In our experiments, while I_G_/I_B_ increased when the Tb^3+^ concentration was increased from 1 to 10 extra wt%, it decreased when the Tb^3+^ concentration was increased from 0.1 to 1 extra wt% (Figure 8), possibly because Tb^3+^ pair formation, clustering, and phase separation played a role in our system [15]. Interestingly, for both excitation wavelengths (λ_exc_ = 193 and 378 nm), I_B_ and I_G_ were comparable for the samples studied (Figure 8).

**Figure 9:**
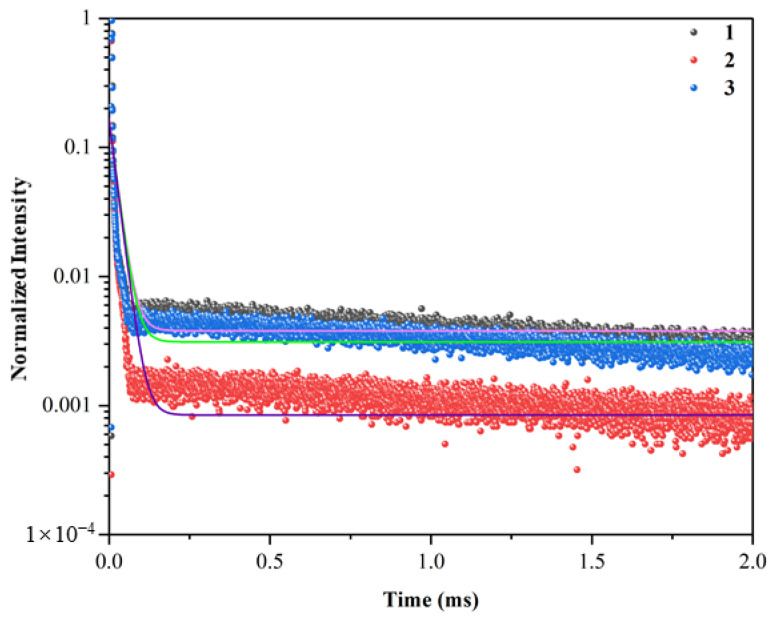
Green luminescence decay curves of the Tb^3+^-activated LTA zeolite-derived boro-aluminosilicate glass samples (each curve was normalized according to the intensity of sample #1). The solid lines represent the results of fitting Equation (1) to the experimental data.

A graph of the green PL decay curves is shown in Figure 9 (λ_exc_ = 378 nm; λ_em_ = 543 nm). The curves did not follow single exponential decays, indicating the presence of a radiationless process due to the energy transfer among active Tb^3+^ ions, caused by either cross-relaxation or a cooperative energy transfer to upper levels [21]. We employed two methods for obtaining the luminescence decay times. In the first method, the following theoretical intensity curve with two exponential decay terms was fitted to the experimental data (Figure 9):

(1) $$I=I_{0}+A1\times exp\left(\frac{-t}{\tau_{1}}\right)+A2\times exp\left(\frac{-t}{\tau_{2}}\right)\;$$

The values for the luminescence decay times (*τ_1_* and *τ_2_*) obtained using this method were similar for the samples and included *τ_1_* = 1.21, 1.21, and 1.20 μs and *τ_2_* = 11.80, 12.28, and 12.00 μs for samples #1, #2, and #3, respectively. In the second method, the mean luminescence decay time (*τ_m_*) was calculated from the following equation [17,22,30,36]

(2) $$\tau_{m}=\frac{\int I\left(t\right)tdt}{\int I\left(t\right)dt}$$

The values obtained for *τ_m_* from this method were 18, 14, and 17 μs for samples #1, #2, and #3, respectively. A decay time on the order of μs is potentially suitable for applications concerning static imaging, UV detection [27], and plasma display panels [28,29,30]. Importantly, these decay times are substantially shorter than those typically obtained for Tb^3+^-doped glass materials, which are on the order of ms (Table 2). The Tb^3+^-doped glass materials listed in Table 2 were based upon calcium aluminosilicate [14,19,21], fluorophosphate [16], fluoroborate [17], zinc phosphate [16,20], LTA zeolite-derived aluminosilicate [20], zinc phosphate [20], zinc fluorophosphate [21], borogermanate [22,29], lead germanate [25], strontium aluminoborate [26], and strontium fluoroaluminate [30] glasses. Of note, in the only other example of LTA zeolite-derived Tb^3+^-doped glass we are aware of [20], the sample was green-emitting and had a decay time of 2.398 ms.

## 4. Conclusions

Colorless, transparent, Tb^3+^-doped, LTA zeolite-derived boro-aluminosilicate glass samples were prepared using the melt quenching method. The emission spectra obtained using two different excitation wavelengths (λ_exc_ = 193 and 378 nm) showed blue and green light emissions with comparable intensities. The green luminescence decay curves were not single-exponential. The mean decay times were 18, 14, and 17 μs for 0.2, 1.0, and 10 extra wt% of Tb^3+^, respectively, and the computed decay times from fitting a theoretical curve with two decay terms were ~1.2 and ~12 μs (irrespective of the Tb^3+^ concentration). Given their relatively fast green luminescence decay times, these synthesized glass materials have potential for applications concerning static imaging, UV detection, and plasma display panels.

## Figures and Tables

**Table 1:** Comparison of nominal and experimental compositions obtained from EDX analysis.

	Nominal (wt%)						Experimental (wt%)						
	Na	Al	Si	O	B	Tb	Na	Al	Si	O	B	Tb	Y
#1	8.5	10.0	10.4	58.7	9.8	0.2	6.2	15.1	25.3	47.9	0	2.4	3.2
#2	8.5	10.0	10.3	58.3	9.8	0.9	6.8	16.95	21.2	45.1	0	3.5	6.5
#3	7.8	9.1	9.5	54.8	9.0	7.7	10.1	12.65	13.8	45.1	0	6.7	11.8

**Table 2:** Green luminescence decay times of various Tb^3+^-doped glasses under excitation with UV light.

Tb^3+^-Doped Glass Material	Composition	Decay Time (ms)	Refs.
LTA zeolite-derived boro-aluminosilicate	xTb_2_O_3_-68(Na_2_O-Al_2_O_3_-SiO_2_)–32B_2_O_3_ (x = 0.2, 1.0 and 10 extra wt%)	τ_1_~1.2 μs, τ_2_~12 μs τ_m_ = 18, 14, 17 μs	Present study
calcium aluminosilicate	47.2CaO-41.3Al_2_O-4.1MgO-7.0SiO_2_-xTb_4_O_7_ (x = 0.04–15) (in wt%) and 33.2CaO-27.7Al_2_O_3_-4.1MgO-34SiO_2_-0.5Tb_4_O_7_ (in wt%)	1.9, 2.3	[14,19]
fluorophosphate	44P_2_O_5_-17K_2_O-(29 − x) SrF_2_-9Al_2_O_3_-x Tb_4_O_7_ (x = 0.1–4) (in mol%)	2.65–2.94	[16]
fluoroborate	(50 − x)B_2_O_3_-20ZnF_2_-30BaF_2_-xTbF_3_ (x = 0.1–4.0) (in mol%)	3.33–4.57	[17]
zinc phosphate	60P_2_O_5_-15ZnO-5Al_2_O_3_-10BaO-10PbO-xTb_2_O_3_ (in mol%) (x = 1.0–5.0) (in wt%)	2.62–2.94	[18]
LTA zeolite-derived aluminosilicate	Na^+^ was ion-exchanged with Tb^3+^ in Na_12_Al_12_Si_12_O_48_	2.398	[19]
calcium aluminosilicate	(100 − x)(58SiO_2_-23CaO-5Al_2_O_3_-4MgO-0NaF in mol%)-xTb_2_O_3_ (x = 0.25–40 in wt%)	2.32–3.38	[20]
zinc phosphate	(100.0 − x)Zn(PO_3_)_2_-xTb_2_O_3_ (x = 0.6–5.0) (in mol%)	2.76–2.97	[22]
zinc fluorophosphate	44P_2_O_5_-17K_2_O-9Al_2_O_3_-(29 − x)ZnF_2_-xTb_4_O_7_ (x = 0.1–2.0) (in mol%)	3.12–3.78	[23]
borogermanate	25B_2_O_3_-40GeO_2_-(35 − x)Gd_2_O_3_-xTb_2_O_3_ (x = 0.25–16) (in mol%)	1.0–1.8	[24]
lead germanate	45PbO-45GeO_2_-9.5Ga_2_O_3_-0.5Tb_2_O_3_ (in mol%)	1.34	[25]
strontium aluminoborate	50B_2_O_3_-15Al_2_O_3_-35-xSrO-xTb_4_O_7_ (x = 0.1–5.0) (in mol%)	2.2–2.6	[26]
strontium fluoroaluminate	70SiO_2_-7Al_2_O_3_-16SrF_2_-7GdF_3_-4TbF_3_ (in mol%)	~3.1	[30]
borogermanate	50GeO_2_-25B_2_O_3_-(25 − x)La_2_O_3_/Gd_2_O_3_-xTb_2_O_3_ (x = 1–4)	1.87–1.97	[31]

## Data Availability

The data of this paper are available on request from the corresponding author.
